# Structural, blood flow and functional changes in the macular area and the expression of aqueous humor factors in myopia

**DOI:** 10.3389/fmed.2024.1335084

**Published:** 2024-07-17

**Authors:** Lu Yang, Honglei Niu, Wencui Sun, Dongchang Zhang, Shuangnong Li, Shaofeng Hao, Minting Wang, Chuan Wang, Junping Hu, Xian Li

**Affiliations:** ^1^Department of Ophthalmology, Shanxi Aier Eye Hospital, Taiyuan, China; ^2^Department of Ophthalmology, Changzhi Aier Eye Hospital, Changzhi, China; ^3^Department of Ophthalmology, Taiyuan Aier Eye Hospital, Taiyuan, China; ^4^Department of Ophthalmology, Shanxi Eye Hospital, Taiyuan, China; ^5^Department of Ophthalmology, Heji Hospital Affiliated to Changzhi Medical College, Changzhi, China

**Keywords:** high myopia, aqueous humor, fixation stability, macular area, ophthalmology

## Abstract

**Objective:**

To compare the macular area parameters and aqueous humor factors between myopia and emmetropia.

**Methods:**

Convenience sampling was used to select patients who visited the Changzhi Aier Eye Hospital’s department of ophthalmology from December 2018 to December 2022 as the study participants. They were divided into three groups according to whether they were diagnosed as mild myopia myopic, highly myopic or not as follows: the mild myopia group (60 cases, 108 eyes), the high myopia group (46 cases, 78 eyes) and the healthy emmetropia group (40 cases, 65 eyes). The differences in the macular integrity (MI) assessment, optical coherence tomography and optical coherence tomography angiography parameters and aqueous humor factors were compared between the three groups.

**Results:**

AL in high myopia group was the highest, and that in emmetropia group was the lowest. The BCVA of mild myopia group was the highest. The RS in the high myopia group were significantly lowest in the three groups (26.42 ± 1.04 vs. 28.34 ± 0.76 vs. 31.92 ± 0.77) (*F* = 5.374, *p* = 0.013). The 63% BCEA, 95% BCEA and MI in the high myopia group were significantly highest (*p* < 0.05). The mean RPE thickness, mean CT and mean RT in the high myopia group were lowest (*p* < 0.05). The blood flow density were lowest in the superficial fovea, paracentral fovea and different subdivisions of the paracentral fovea in the high myopia group (*p* < 0.05). The VEGF concentration in the aqueous humor of the high myopia group was lowest (25.62 ± 17.43 vs. 32.45 ± 24.67 vs. 64.37 ± 21.14) (*F* = 9.237, *p* < 0.001). The MMP-2 concentration was highest (483 ± 201.48 vs. 410 ± 142.37 vs. 386 ± 154.34) (*F* = 5.542, *p =* 0.018). The VEGF concentration in the aqueous humor factor was negatively correlated with the AL in the myopia group (*r* = −0.438, *p* = 0.002), the MMP-2 concentration was positively correlated with the AL (*r* = 0.484, *p* = 0.010).

**Conclusion:**

Patients with high myopia showed decreased retinal light sensitivity, fixation stability, superficial blood flow density and retinal thickness compared with people with emmetropia. A decreased VEGF concentration and increased MMP-2 concentration in the aqueous humor factor have potential associations with the development of high myopia.

## 1 Introduction

High myopia is a serious form of myopia. According to statistics, the myopia rate in young adults in the United States has increased by a factor of eight during the past 30 years, and the situation is not optimistic in the young Asian population (1). It is estimated that the number of patients with high myopia worldwide will reach 938 million by 2050, accounting for 9.8% of the world population (2). High myopia can lead to myopic retinopathy, which often manifests as lacquer crack formation, macular schisis, posterior scleral staphyloma, macular hemorrhage, retinal detachment, retinochoroidal atrophy, etc., which may cause permanent visual impairment or blindness (2, 3). The natural course of early pathological myopia is slow, and the best corrected visual acuity (BCVA) is often not affected (4). BCVA changes when pathological myopia is complicated by maculopathy, and the visual impairment caused is usually irreversible. There is no effective treatment, and some investigators have studied the structure and function of high myopia separately, but fewer have assessed the structure, blood flow, function and aqueous humor factor pathway involved in the development of high myopia.

The vascular endothelial growth factor (VEGF) and pigment epithelium-derived factor (PEDF) in the aqueous humor are secreted and produced by the retinal pigment epithelium (RPE), and they are involved in regulating intraocular vessels (5). The matrix metalloproteinases 2 (MMP-2) has also been shown to be present in Bruch’s membrane (6) and is one of the metalloproteinases involved in degrading the RPE basement membrane (BM) and choroid (7). Previous studies have shown alterations in the levels of these factors in the aqueous humor of myopic individuals (8, 9). Increased VEGF levels have been associated with vascular changes, while changes in PEDF and MMP-2 may affect retinal structure and matrix turnover (9). The investigation of the above aqueous humor factors may provide further insight into the mechanisms underlying the development of high myopia. Therefore, this study comprehensively analyzed the changes in the ocular characteristics of high myopia and elucidated the changes through various parameters and aqueous humor factors during the development of high myopia by optical coherence tomography (OCT), optical coherence tomography angiography (OCTA), macular integrity assessment (MAIA) microperimetry and enzyme-linked immunosorbent assay. This study hopes to make a preliminary prediction for people with pathological changes in high myopia and offer guidance in early clinical intervention.

## 2 Participants and methods

### 2.1 Participants

A retrospective study was conducted to select patients who visited the Changzhi Aier Eye Hospital from December 2018 to December 2022 using convenience sampling (for its simplicity and practicality). The subjects were divided into three groups according to whether they were diagnosed as mild myopic, highly myopic or not as follows: the mild myopia group (108 eyes in 60 cases), the high myopia group (78 eyes in 46 cases) and the healthy emmetropia group (65 eyes in 40 cases). The inclusion criteria for the mild group was as follows: −6.0 D < refraction < −0.5 D. The inclusion criteria for the high myopia group were as follows: (1) refraction ≤ −6.0 D, BCVA ≥0.8; (2) axial length (AL) ≥ 26.5 mm; (3) intraocular pressure 11–21 mmHg. The inclusion criteria for the healthy emmetropia group were as follows: (1) −0.5 D ≤ refraction ≤0.5 D; (2) uncorrected visual acuity ≥0.8; (3) normal by OCT and fundus examination. The exclusion criteria were as follows: (1) patients with cataracts, glaucoma, ocular trauma, history of ocular surgery and other eye diseases that may affect the results of instrument testing; (2) patients with active inflammation in the eye, which is accompanied by other retinal or vitreous diseases; (3) patients treated with drugs that had an impact on blood flow function within the past 2 weeks; (4) other systemic diseases, such as high blood pressure, diabetes, and infections. All patients and their families cooperated with the trial process and signed an informed consent form, and this study was approved by the Changzhi Aie Eye Hospital Ethics Committee [Approval number: Changzhi Aie Eye Hospital Ethics (2022) No. 2].

### 2.2 Methods

All participants underwent routine ophthalmic examinations, including uncorrected visual acuity and BCVA, AL, intraocular pressure and dilated fundus examination.

#### 2.2.1 Determination of retinal thickness in each layer

**Figure 1:**
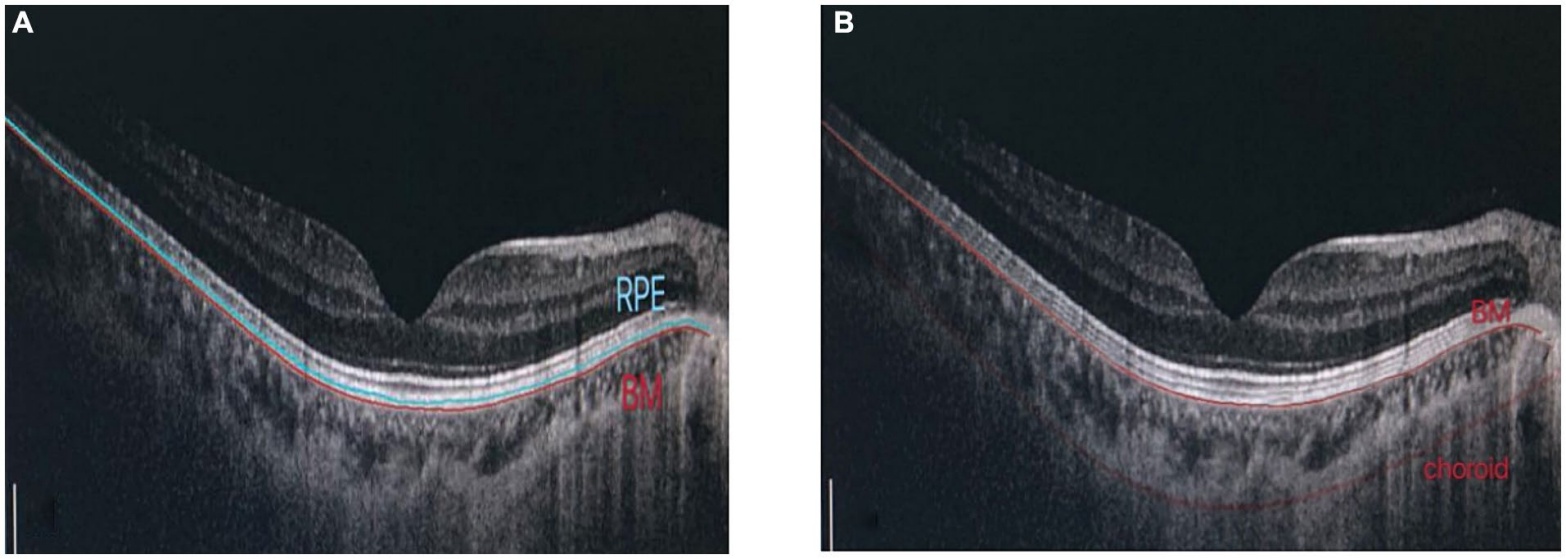
Determination of RPE thickness, choroidal thickness: **(A)** RPE layer is defined as the thickness from RPE to BM layer. **(B)** Manually adjust choroidal divider.

Retinal pigment epithelium thickness, choroidal thickness (CT) and retinal thickness (RT) of the fovea were measured using OCT (OCT, Heidelberg, Germany). Macular RT was scanned using the enhanced depth imaging mode of SD-OCT. All images were analyzed using the Heidelberg Eye Explore 1.9.10.0 software, where the RPE layer (10) was defined as the thickness from the RPE to the BM layer. CT was defined as the vertical distance from the outer edge of the RPE layer to the inner edge of the sclera. After the system automatically calculated RPE thickness, CT was checked by manually moving the retinal segmentation line to the choroid (Figure 1). It was presented as nine areas with the Early Treatment of Diabetic Retinopathy Study (ETDRS) form by the self-contained, built-in mapping software. The average thickness of each layer was calculated from the average thickness of nine measurement points in the superior, inferior, nasal, temporal and foveal regions with a foveal diameter of 3–6 mm.

#### 2.2.2 Densitometry of superficial blood flow in the macular area

**Figure 2:**
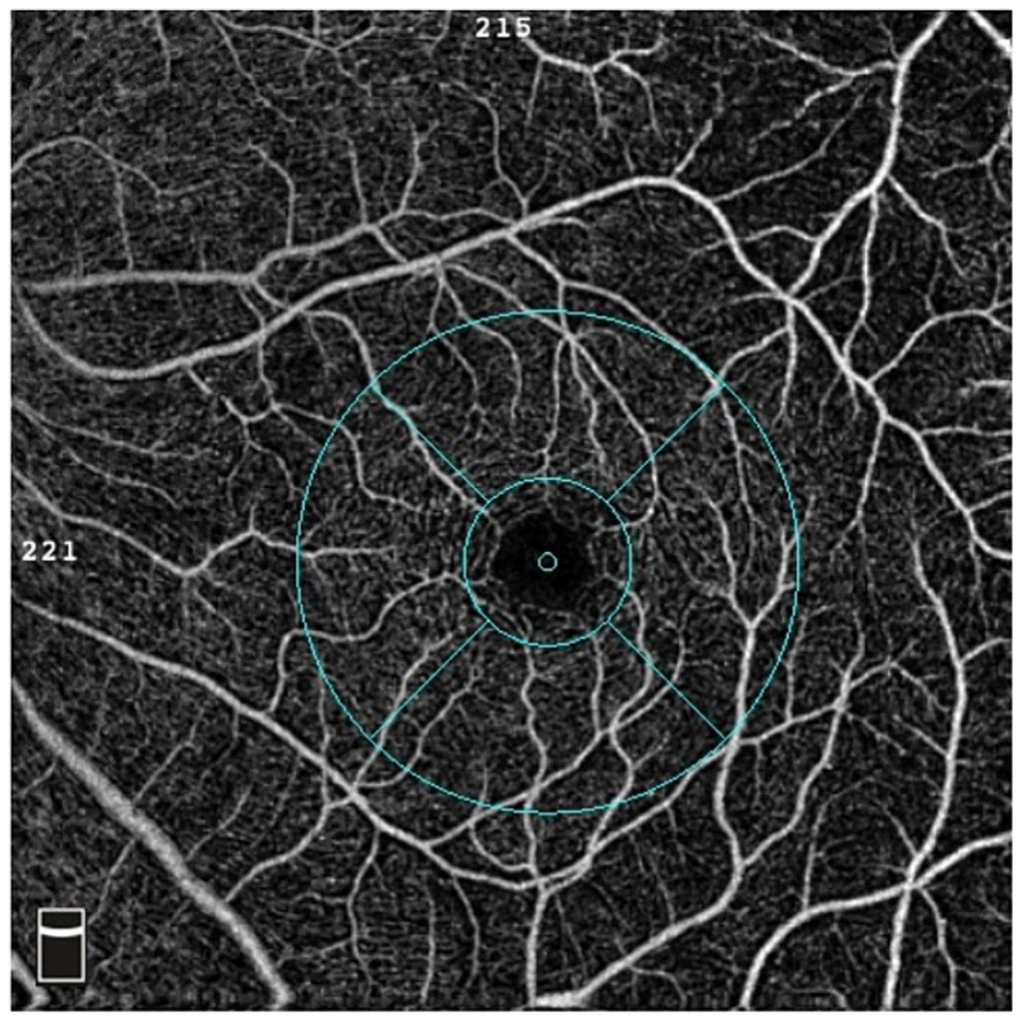
OCTA software automatically measures and partitions the blood flow density in the macular area.

Optical coherence tomography angiography (Optpvue, America) was used to select the macular blood flow imaging program, palisade scanning was centered on the fovea with a scanning range of 3 mm × 3 mm and the horizontal and vertical phases were each scanned once. According to the principle of motion correction, macular microflow maps were obtained after the correction of the eye movement, with an image resolution of 304 px × 304 px and a scanning time of 2.9 s in each phase. Microflow maps could be obtained from retinal superficial microflow maps by automatic stratification (Figure 2).

#### 2.2.3 Macular retinal light sensitivity, fixation stability, and macular integrity determination

**Figure 3:**
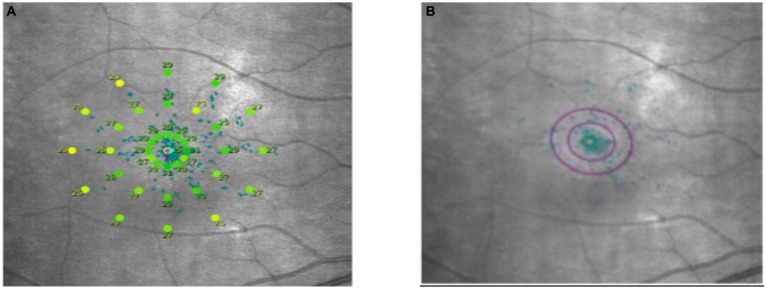
Microperimetry illustration. **(A)** Microperimetry measures the 10° central area of the macula. **(B)** 63 and 95% hyperbolic ellipse area to assess fixation stability.

**Figure 4:**
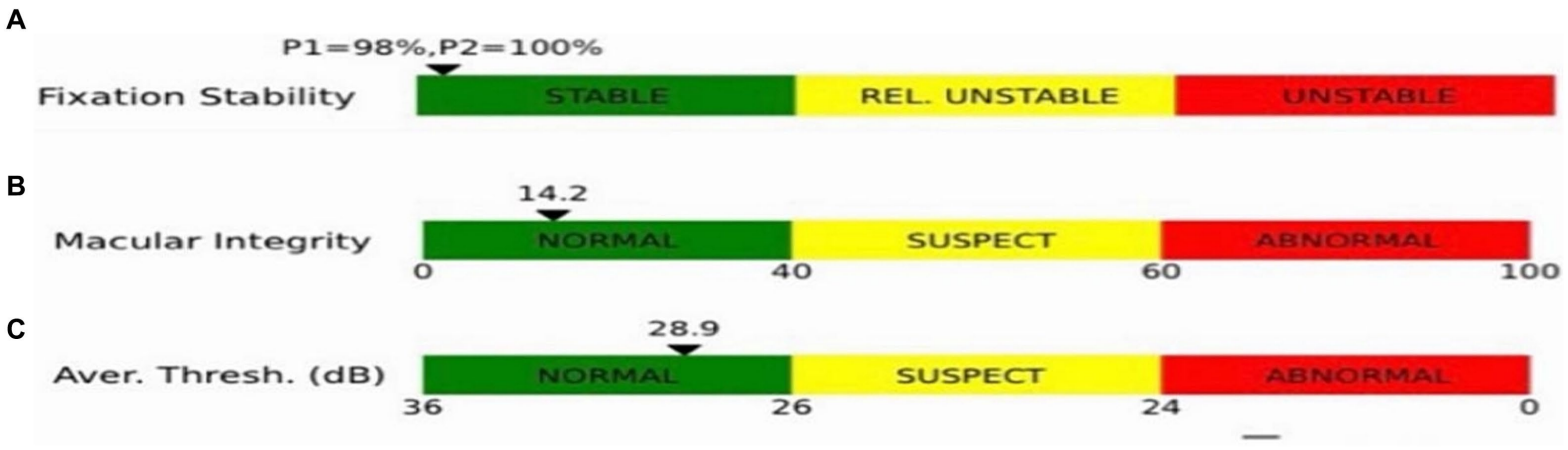
Indices shown in the microfield. **(A)** Indicators of fixation stability (P1, P2). **(B)** Macular integrity indicator. **(C)** The smaller the value of the retinal light sensitivity index, which resides in the green bar, the better the macular function; the larger the value, residing in the red bar, the poorer the macular function, with yellow in between.

Macular integrity assessment (CenterVue, Italy) microperimetry was used to detect retinal light sensitivity (RS), fixation stability (FS) and the macular integrity (MI) index within 10° of the macular area of the eye. All participants did not require mydriasis, and the high myopia and the healthy emmetropia group were tested with corrective lenses at best visual acuity. The participants were habituated in a dark room for 5 min with a detection zone of concentric circles at 1°, 3°, and 5° in the fovea and a cursor set using Goldmann III visual target with a maximum stimulus luminance of 1,000 asb and a minimum stimulus luminance of 0.25 asb. This corresponded to RS of 0 dB and 36 dB, respectively, cursor duration of 200 ms and white background luminance of 4 asb. FS analysis was performed by automatically calculating the bivariate contour ellipse area (BCEA) at 63 and 95% of the fixation range by the system, and the MI index was recorded (Figures 3, 4).

#### 2.2.4 Determination of the aqueous humor factor concentration by enzyme-linked immunosorbent assay

Undiluted aqueous humor was collected from patients from the high myopia and emmetropia groups, and samples were stored in sterile reagent tubes in a −80° freezer until they were processed for use. The concentrations of VEGF, PEDF and MMP-2 were determined by double-antibody sandwich enzyme-linked immunosorbent assay (kit manufacturer: Shanghai Enzyme-Linked Biotechnology Co., Ltd.), and all processes were performed in strict accordance with the instructions. Briefly, after the samples were prepared, the samples were added to the bottom of the ELISA plate for incubation. Incubation conditions were 37°C for 30–60 min. After the end of incubation, washing was performed using washing solution. Biotin-labeled antibodies were subsequently added and incubated at 37°C for 60 min. At the end of incubation, washing was performed using washing solution. Following washing, staining was performed using TMB chromogenic substrate for 10 min. At the end of staining, stop solution was added for staining termination. Finally, the result measurement was performed using a microplate reader.

### 2.3 Statistical analysis

The SPSS 22.0 analysis software was used. The data information of each group conformed to normal distribution, the measurement data were statistically described using mean ± standard deviation (x― ± S), analysis of variance was used for comparison among three groups, and LSD was used for pairwise comparison. Furthermore, the correlation between each index in myopia groups (the mild myopia group and the high myopia group) was analyzed using the Pearson correlation analysis, and *p* < 0.05 was considered statistically significant.

## 3 Results

### 3.1 Comparison of clinical characteristics of the participants

The emmetropia group (*n* = 40, 65 eyes) consisted of 18 men and 22 women. The mild myopia group (*n* = 60, 108 eyes) consisted of 34 men and 26 women. The high myopia group (*n* = 46, 78 eyes) consisted of 21 men and 25 women. In this trial, there were significant difference in AL and BCVA in the three groups (*p* < 0.05). AL in high myopia group was the highest, and that in emmetropia group was the lowest. The BCVA of mild myopia group was the highest, and there was no difference between high myopia group and emmetropia group (see Table 1).

**Table 1:** Comparison of subject clinical characteristics.

Group	Emetropia group (*n* = 65)	Mild myopic group (*n* = 108)	High myopia group (*n* = 78)	*F* value	*p* value
Age	27.94 ± 8.17	28.71 ± 10.71	30.04 ± 8.19	2.234	0.094
Sex (male/female)	18/22	34/26	21/25	1.814	0.404
IOP (PMHg)	15.49 ± 3.36	15.87 ± 3.34	16.43 ± 2.76	1.247	0.159
AL (PM)	22.24 ± 0.41	24.16 ± 0.66^*^	26.06 ± 0.74^*#^	7.782	0.003
Mean BCVA	0.89 ± 0.17	0.94 ± 0.13^*^	0.85 ± 0.11^#^	4.812	0.028

^*^Compared with the emetropia group, the difference was statistically significant, *p* < 0.05. ^#^Compared with the Mild myopic group, the difference was statistically significant, *p* < 0.05. AL, Axial Length; IOP, Intraocular Pressure; BCVA, Best-Corrected Visual Acuity.

### 3.2 Comparison of RS, fixation stability and the macular integrity index between the two groups

The RS in the high myopia group were significantly lowest in the three groups (26.42 ± 1.04 vs. 28.34 ± 0.76 vs. 31.92 ± 0.77) (*F* = 5.374, *p* = 0.013). The 63% BCEA, 95% BCEA and MI in the high myopia group were significantly highest (*p* < 0.05) (see Table 2).

**Table 2:** Comparison of RS, FS and MI between two groups.

	Emmetropia group	Mild myopic group	High myopia group	*F* value	*p* value
RS (dB)	31.92 ± 0.77	28.34 ± 0.76*	26.42 ± 1.04^*#^	5.374	0.013
63% BCEA (Deg)	0.68 ± 0.45	0.86 ± 0.42^*^	1.94 ± 0.28^*#^	4.141	0.030
95% BCEA (Deg)	1.87 ± 0.59	2.23 ± 0.74^*^	3.68 ± 1.55^*#^	3.237	0.031
MI	10.94 ± 4.86	19.37 ± 6.74^*^	30.24 ± 5.83^*#^	9.470	<0.001

^*^Compared with the emetropia group, the difference was statistically significant, *p* < 0.05. ^#^Compared with the Mild myopic group, the difference was statistically significant, *p* < 0.05. RS, retinal light sensitivity; BCEA, hyperbolic ellipse area; MI, macular integrity.

### 3.3 Comparison of retinal pigment epithelium thickness, choroidal thickness and retinal thickness between the two groups

The mean RPE thickness, mean CT and mean RT in the emmetropia group were highest in the three groups, and the difference was statistically significant (see Table 3).

**Table 3:** Comparison of retinal pigment epithelium thickness, choroidal thickness and retinal thickness between the two groups.

	Emmetropia group	Mild myopic group	High myopia group	*F* value	*p* value
Mean RPE (μm)	27.88 ± 1.62	25.16 ± 1.34^*^	23.01 ± 1.50^*#^	13.414	0.004
Mean CT (μm)	319.78 ± 50.12	284.64 ± 40.83^*^	219.12 ± 42.57^*#^	29.412	<0.001
Mean RT (μm)	337.98 ± 23.71	324.08 ± 13.87^*^	302.13 ± 18.05^*#^	25.381	<0.001

^*^Compared with the emetropia group, the difference was statistically significant, *p* < 0.05. ^#^Compared with the Mild myopic group, the difference was statistically significant, *p* < 0.05. RPE, the retinal pigment epithelium; CT, choroidal thickness; RT, retinal thickness.

### 3.4 Comparison of superficial blood flow density in the macular area between the two groups

The blood flow density were lowest in the superficial fovea, paracentral fovea and different subdivisions of the paracentral fovea in the high myopia group, with statistically significant differences (*p* < 0.05) (see Table 4).

**Table 4:** Comparison of macular blood flow density between the two groups.

	Emmetropia group	Mild myopic group	High myopia group	*F* value	*p* value
Surface fovea (%)	24.81 ± 1.49	22.22 ± 1.31^*^	20.84 ± 1.78^*#^	18.212	<0.001
Superficial parafovea (%)	53.48 ± 2.42	51.12 ± 2.09^*^	49.35 ± 3.08^*#^	12.225	<0.001
Superficial temporal (%)	50.43 ± 1.62	49.24 ± 1.51	48.33 ± 2.86^*^	6.348	0.021
Superficial upper side (%)	52.08 ± 2.61	51.24 ± 2.87	49.69 ± 3.67^*#^	4.934	0.026
Superficial nasal (%)	51.09 ± 2.34	50.37 ± 2.85	49.64 ± 2.78^*^	5.567	0.032
Superficial lower side (%)	51.84 ± 4.55	50.48 ± 4.17	47.11 ± 4.32^*^	3.25	0.024

^*^Compared with the emetropia group, the difference was statistically significant, *p* < 0.05. ^#^Compared with the Mild myopic group, the difference was statistically significant, *p* < 0.05.

### 3.5 Comparison of aqueous humor factors between the two groups

The VEGF concentration in the aqueous humor of the high myopia group was lowest in the three groups (25.62 ± 17.43 vs. 32.45 ± 24.67 vs. 64.37 ± 21.14), and the difference was statistically significant (*F* = 9.237, *p* < 0.001). The MMP-2 concentration was highest (483 ± 201.48 vs. 410 ± 142.37 vs. 386 ± 154.34), and the difference was statistically significant (*F* = 5.542, *p =* 0.018). However, there were no significant difference in PEDF (see Table 5).

**Table 5:** Comparison of aqueous humor factors between the two groups.

Aqueous factor	Emmetropia group	Mild myopic group	High myopia group	*F* value	*p* value
VEGF (pg/ml)	64.37 ± 21.14	32.45 ± 24.67^*^	25.62 ± 17.43^*#^	9.237	<0.001
PEDF (ng/ml)	3.15 ± 1.23	3.32 ± 1.38	3.83 ± 2.27	3.534	0.061
MMP-2 (pg/ml)	386 ± 154.34	410 ± 142.37	483 ± 201.48^*^	5.542	0.018

^*^Compared with the emetropia group, the difference was statistically significant, *p* < 0.05. ^#^Compared with the Mild myopic group, the difference was statistically significant, *p* < 0.05. VEGF, The vascular endothelial growth factor; PEDF, pigment epithelium-derived factor; MMP-2, The matrix metalloproteinases 2.

### 3.6 Correlation analysis between the axial length and various parameters

With the extension of the AL, FS, RT in each layer and superficial blood flow density decreased (*p* < 0.05), and the differences were statistically significant. The VEGF concentration in the aqueous humor factor was negatively correlated with the AL in the myopia group (*r* = −0.438, *p* = 0.002), and the difference was statistically significant. The MMP-2 concentration was positively correlated with the AL (*r* = 0.484, *p* = 0.010), and the difference was statistically significant (see Table 6).

**Table 6:** Correlation between various indicators and axial length, aqueous humor factors.

		AL	VEGF	PEDF	MMP-2
63% BCEA	R	0.231	0.074	0.182	0.078
	*p* value	0.157	0.061	0.160	0.080
95% BCEA	R	0.321	0.296	0.187	0.319
	*p* value	0.102	0.200	0.304	0.404
Mean RPE (μm)	R	0.394	0.355	0.401	0.090
	*p* value	0.095	0.362	0.417	0.295
Mean RT (μm)	R	0.307	0.142	0.145	0.122
	*p* value	0.413	0.408	0.397	0.167
Mean CT (μm)	R	0.366	0.080	0.373	0.209
	*p* value	0.148	0.272	0.381	0.088
Parafoveal SVD (%)	R	0.393	0.145	0.263	0.430
	*p* value	0.084	0.076	0.240	0.124
Foveal SVD (%)	R	0.372	0.240	0.367	0.227
	*p* value	0.084	0.410	0.330	0.118
VEGF (pg/ml)	R	**−0.438**	–	0.103	0.324
	*p* value	**0.002**	–	0.167	0.196
PEDF (ng/ml)	R	0.293	0.089	–	0.310
	*p* value	0.084	0.430	–	0.258
MMP-2 (pg/ml)	R	**0.484**	0.148	0.394	–
	*p* value	**0.010**	0.126	0.228	–

VEGF, vascular endothelial growth factor; RPE, retinal pigment epithelium; MMP-2, matrix metalloproteinases 2.

## 4 Discussion

This study analyzed changes in RT, CT, and retinal pigment epithelial thickness measured by OCT, superficial blood flow density measured by OCTA and retinal sensitivity, FS and MI measured by MAIA microperimetry in the high myopia and emmetropia groups. Overall, RT, RS, FS and blood flow density decreased in patients with high myopia.

Previous studies have (11–15) demonstrated that RT, CT and retinal pigment epithelial thickness decrease with AL extension in high myopia. Ucak et al. (16) and Fan et al. (17) proposed a negative correlation between axial elongation and superficial and deep blood flow density, with passive retinal elongation and decreased blood flow density with axial elongation. Wang et al. (18) concluded that decreased macular sensitivity in high myopia may be related to changes in the density distribution of cones and RT. Zhu et al. (19) also found a good correlation between FS and AL in high myopic eyes, which decreased with AL extension. The conclusions reached by the above investigators are consistent with this study’s results. In addition, it was found that the MI index was significantly higher in the high myopia group than in the emmetropia group. The higher the MI index, the greater the possibility of abnormal visual function. There have been few previous studies on MI. The results of this study suggest that macular function tends to deteriorate in patients with high myopia. Patients should be closely monitored, and attention should be paid to changes in their conditions. Intervention measures should also be taken as early as possible to reduce further macular lesions.

Many growth factors are found in ocular media, which regulate lens cell behaviors, including PEDF, VEGF, and MMP-2 (20). Increased PEDF or decreased VEGF expression inhibits angiogenesis, whereas increased VEGF promotes angiogenesis, and MMP-2 is one of the proteins produced directly downstream to VEGF (21, 22). The changes of these growth factors leading to myopia may be related to the mechanism of oxidative stress (9). The levels of these factors detected in this study are similar to previously reported results, which further validates our findings and strengthens our understanding of the role of these factors in the development of high myopia (23). The results of this study showed that the MMP-2 concentration in aqueous humor increased in the high myopia group compared with the emmetropia group and was positively correlated with the AL. The MMP-2 is present in Bruch’s membrane and is involved in the degradation of the extracellular matrix. It is also highly expressed in the vitreous cavity (24). Wong et al. (25) suggested that high concentrations of MMP-2 may be the cause of axial elongation caused by the abnormal rupture of Bruch’s membrane extracellular matrix. As the AL continues to lengthen, RPE –Bruch’s membrane– choroidal complex is affected, which subsequently leads to lacquer cracks, the loss of Bruch’s membrane and the thinning of CT. Jia et al. (26) pointed out in their study that the MMP-2-enhanced activity degrades the scleral extracellular matrix and collagen, leading to scleral weakness, which subsequently causes axial elongation and myopia development. Therefore, the MMP-2 can be used as a monitoring factor. When the MMP-2 concentration in the aqueous humor increases, it may be developing into high myopia, and relevant measures should be taken in time for prevention.

The results of this study showed that VEGF concentration in the aqueous humor of the high myopia group decreased compared with the emmetropia group, and VEGF concentration decreased with the extension of the AL, while PEDF concentration was not statistically different from that of the emmetropia group and was not correlated with the AL. The study results of Shin et al. (23) are consistent with this study’s conclusions, and they propose that high myopia disrupts VEGF/PEDF balance in RPE. VEGF and PEDF are secreted by retinal pigment epithelial cells (5), and according to the conclusions drawn, it can be seen that degenerative changes in retinal pigment epithelial thickness in high myopia and the degeneration of the retinal pigment epithelial layer lead to photoreceptor degeneration (27), which subsequently affects visual function. Most investigators believe that the concentration of VEGF should be consistent with the intravitreal concentration, so anti-VEGF drugs are used in patients with high myopia that is complicated by choroidal neovascularization. This may be because the AL of high myopia eyes is prolonged, the eyeballs become larger and the VEGF concentration decreases with the AL extension, which is related to the dilution of the concentration in the aqueous humor (28). However, the dilution effect cannot explain the correlation between the levels of other aqueous humor factors and the AL, and the specific mechanism needs to be further explored.

The shortcomings of this study are that the high myopia group was not divided into pathological and simple groups, and fewer aqueous factors were selected. Secondly, the sampling method in this study is convenience sampling, and a relatively small sample size may further reduce the validity of evidence. In future studies, we will use random sampling method for verification. In addition, in terms of sample collection, the samples for biomarker detection are mainly taken from the anterior segment. Since changes in myopia are mainly associated with changes in the posterior pole, this may result in a less pronounced relationship between VEGF or MMP-2 and high myopia. In future studies, we will take samples from the posterior chamber to detect biomarkers. The sample size is expected to expand in a future study with further groups to investigate the levels of multiple aqueous factors for clinical studies.

## 5 Conclusion

In summary, RT, blood flow density and visual function are impaired in patients with high myopia, while our findings suggest a potential association between concentrations of VEGF and MMP-2 in the aqueous humor and the development of high myopia. These indicators can be used as monitoring factors, and clinical intervention can be taken in time when there are changes to control their development to high myopia and prevent further development to the irreversible stage of pathological myopia.

## Data availability statement

The original contributions presented in the study are included in the article/supplementary material, further inquiries can be directed to the corresponding authors.

## Ethics statement

The studies involving humans were approved by ethics committee of Shanxi Aier Eye Hospital. The studies were conducted in accordance with the local legislation and institutional requirements. The participants provided their written informed consent to participate in this study.

## Author contributions

LY: Conceptualization, Methodology, Writing – original draft, Writing – review & editing. HN: Conceptualization, Writing – original draft, Writing – review & editing. WS: Conceptualization, Writing – original draft, Writing – review & editing. DZ: Conceptualization, Writing – original draft, Writing – review & editing. SL: Methodology, Writing – original draft, Writing – review & editing. SH: Data curation, Writing – original draft, Writing – review & editing. MW: Data curation, Writing – original draft, Writing – review & editing. CW: Data curation, Writing – original draft, Writing – review & editing. JH: Formal analysis, Writing – original draft, Writing – review & editing. XL: Formal analysis, Writing – original draft, Writing – review & editing.
